# Role of Mesotherapy in Musculoskeletal Pain: Opinions from the Italian Society of Mesotherapy

**DOI:** 10.1155/2012/436959

**Published:** 2012-05-13

**Authors:** Massimo Mammucari, Antonio Gatti, Sergio Maggiori, Alessandro F. Sabato

**Affiliations:** ^1^The Italian Society of Mesotherapy, Rome, Italy; ^2^Emergency Care, Department of Intensive Care, Pain Medicine, and Anaesthesiology, Tor Vergata Polyclinic, University of Tor Vergata, 00133 Rome, Italy

## Abstract

Mesotherapy is the injection of active substances into the surface layer of the skin. This method allows a slower spread, higher levels, and longer lasting effects of drugs in the tissues underlying the site of injection (skin, muscle, and joint) compared with those following intramuscular injection. This technique is useful when a local pharmacological effect is required and relatively high doses of drug in the systemic circulation are not. Mesotherapy should only be undertaken following a complete clinical workup and subsequent diagnosis. Encouraging results have been reported in randomized, controlled clinical trials and in observational studies involving patients with various forms of musculoskeletal pain. Recommendations by experts from the Italian Society of Mesotherapy for appropriate use of mesotherapy in musculoskeletal pain and an algorithm for treating localized painful conditions are provided.

## 1. Introduction

Mesotherapy is a technique used to inject active substances into the superficial layer of the skin [1–4]. The objective of this type of administration is to modulate the pharmacokinetics of the injected substance and to prolong the pharmacological effects at a local level. One of the main advantages of mesotherapy is that a local pharmacological effect can be obtained without the need for high systemic concentrations [4, 5]. Our group has previously demonstrated that intradermal injections of small amounts of active substance where the injection site corresponds to the area of the pathological condition—for example, in lower back pain—may provide clinical benefits where other therapies are not available/not effective or cannot be used for whatever reason [5]. In addition, intradermal administration of active substances in combination with other systemic therapies can produce synergistic effects, and as a result mesotherapy may have dose-sparing effects [5].

The correct use of mesotherapy requires clinical and pharmacological skills. It is vital that the procedure is carried out using strictly controlled aseptic techniques and that proper hygiene and sterilization procedures are employed. As a result of the use of this procedure by nonmedical personnel, the failure to comply with hygiene standards, incorrect administration techniques and the misuse of drugs (medications mixed together), a number of adverse events have been reported in the medical literature, in particular due to the lack of aseptic conditions [6]. Furthermore, the lack of randomized clinical trials has raised certain doubts on the validity of this technique [7, 8]. In this review we present a critical analysis of the efficacy and tolerability of mesotherapy in patients with painful musculoskeletal conditions.

## 2. Reasons to Consider Mesotherapy in Pain Management

Pain is an “unpleasant sensory and emotional experience associated with actual or potential tissue damage, or described in terms of such damage” [9]. Acute pain is interpreted as an alarm signal related to actual or potential tissue damage—when pain persists it can become a serious condition in its own right [10]. Usually, pain is caused by stimuli approaching or exceeding harmful intensity, but in the case of prolonged pain the sensitive feedback system is altered, and microglia cells are activated [11, 12]. Even if this hypothesis is not confirmed at clinical level, it could explain the relationships between the lack of inflammatory substances and the chronic pain and the central nervous system pain control failure. Chronic pain is defined as pain that persists for longer than three months [13]. In patients with arthritis or other musculoskeletal conditions, pain is frequently triggered by inflammation of peripheral tissues (nociceptive pain), but it is also associated with a lesion (or dysfunction) of the nerve pathways (neuropathic pain). More often, nociceptive pain and neuropathic pain coexist particularly in patients with chronic back pain [14]. Unfortunately, there is no universally recognised standard of care as there is a number of distinct pathological mechanisms of pain (acute or chronic) as well as a wide range of therapeutic options to manage patients with chronic pain including pharmacological and interventional treatments physical, psychological, complementary and alternative medicine approaches [15]. In Europe nonsteroidal anti-inflammatory drugs (NSAIDs) are the first-line therapy in the majority of patients with musculoskeletal pain in conjunction with nonpharmacological therapies, such as exercise, physiotherapy, acupuncture and herbal-based preparations [16]. The high frequency of adverse events with NSAIDs—gastrointestinal toxicity, renal dysfunction, cardiovascular complications, and the risk of drug–drug interactions, particularly in older patients with comorbidities—in part explains the increasingly widespread use of “alternative” treatments [17]. Local pharmacological therapy, if effective and well tolerated, represents an acceptable alternative to systemic NSAIDs [18, 19].

Mesotherapy consists of a series of “microinjections” of drug/active substance into the dermis using short needles where the needle is positioned at an appropriate angle depending on the thickness of the skin. We suggest using a single needle, 4 mm (27 gauge) or 13 mm (30 or 32 gauge), positioned at 30–45 degree with respect to the skin surface. In general, 0.10–0.20 mL of product is used and injection points are usually 2 or 3 cm apart. If large areas are to be treated, the drug can be diluted, but this reduces the dosage, and, therefore, additional or more frequent injections are necessary. Following injection, the drug slowly reaches the underlying tissues achieving concentrations higher than those obtained with intramuscular administration [5]. Interestingly, some authors consider mesotherapy as an intra- or subcutaneous technique; however, subcutaneously administered drugs may have different pharmacokinetics (diffusion and distribution) and as a result different onset and duration of activity depending on the site of injection [20, 21]. For example, plasma glucose levels vary depending on the subcutaneous site of injection—abdomen, arm, or leg—due to the level of absorption at the various injection sites [22]. In contrast, injection into the superficial layer of the skin (intradermal) allows slow diffusion of the drug into the tissues underlying the site of injection. Sodium ketoprofen levels in skin, muscles, and joints following local intradermal or intramuscular (IM) have been measured in preclinical studies, and results show higher concentrations of the drug in skin, local muscles, and joints (corresponding the site of injection) following intradermal administration compared with following IM injection and these levels which remain high for longer than following IM administration (Table 1) [5, 23]. These results were confirmed with the intradermal inoculation of procaine and penicillin G [24, 25]. Similar results were demonstrated in human studies following intradermal injection (up to 4 mm), and interestingly results confirmed that when a drug is injected at a depth of more than 10 mm it remains for a short time in the surrounding tissues and reaches the systemic circulation rapidly [6].

To confirm that LIT administration provides prolonged concentration of drugs to local tissues, a study was conducted to compare the immunogenic properties of the tetanus toxoid after intradermal and IM administration [26]. This preclinical study showed that more potent immunological responses (primary and secondary) were elicited after intradermal injection. The authors suggest that the unique capacity of dermis to respond to external stimuli together with the increased distribution of antigen at the area of inoculation may go some way to explain these findings and have shown the way to a renewed interest in using intradermal administration of human vaccines to reduce antigen concentration (dose sparing) and the possibility of reducing the need for adjuvants (drug sparing) [27].

A recently published study reported that the administration of recombinant human follicle-stimulating hormone (rhFSH) injected into the abdominal skin at a depth of 1-2 mm, instead of using subcutaneous (10–13 mm) administration, extended the absorption of FSH. These data confirm the many potential clinical advantages of using intradermal injection—dose reduction, reduced number of injections needed to maintain elevated FSH levels, and reduced risk of adverse events [28]. The superficial layer of the skin appears to suggest a sort of an innate “slow-release system” (to be demonstrated with other preclinical and clinical studies), and it is interesting to note that the terminology “local intradermal therapy” is used to highlight the fact that it modulates the absorption and diffusion of drugs at a local level [5], even if till today studies were not produced with the technique of microdialysis.

However, the pharmacological effects of intradermal administration do not account entirely for the observed clinical benefits of mesotherapy. It is thought that “microdoses” of active substances produce a mechanical distention of the surrounding tissues and sensitive fibres. The needle prick activates the cutaneous and subcutaneous receptors (reflex effect), and it is thought that endorphins levels actually increase after the introduction of the needle, but this hypothesis was not confirmed. Furthermore, interactions between the microvascular system and the immune cells in the dermis may play a role in the clinical benefits [29, 30]. Finally, we can argue that the clinical benefits reported by patients treated with analgesic drugs by intradermal injection may be the result of a series of “mesodermic phenomena” that are commonly referred to as mesotherapy.

## 3. Clinical Trials of Mesotherapy in Musculoskeletal Pain

The first series of open studies conducted in patients with musculoskeletal pain conditions—including arthritis, neck pain, lower back pain, and tendinopathy—showed promising results in termss of pain reduction of at least 50% compared to baseline (Table 2) [31–46]. Positive results in term of reduction of pain and complete recovery were also reported in clinical studies conducted in professional and amateur athletes with posttraumatic pain (Table 3) [47–52]. In randomized and controlled trials, clinical benefits were reported in patients with low back pain, with cervicobrachialgia, and with calcifying tendinitis of the shoulder (Table 4) [53–57]. Confirming those previously reported by other authors showing pain relief and reduction of calcification [39, 43, 58, 59]. Interestingly, better results were reported when mesotherapy was combined with other therapies, for example, transcutaneous electric nerve stimulation (TENS) and laser or dynamic therapy [45, 54, 60–62]. Preliminary positive data have also been obtained in painful orodental conditions [63–65].

## 4. Adverse Events and Local Reactions to Mesotherapy

While there is a great deal of data showing that this technique is well tolerated (Tables 2–4), transitory and reversible adverse reactions (allergic reactions, ecchymosis, and urtica) have been described after mesotherapy [4]. Mesotherapy can cause mild discomfort when the needle is introduced, and this is more common in sensitive patients. For this reason it is recommended that the needle should be inserted quickly and gently and that the contents of the syringe are emptied slowly. The different pH of some medications can cause pain during injection, and adjustment of pH with NaHCO_3_ has been suggested [66]. However, it is not clear if the painful stimulus caused by microinjections represents an artificial painful impulse (pain scrambler) that participates in the interruption of transmission of “pain” [67–69]. Other local transitory effects (itching, hypersensitivity, discomfort, and irritation), probably due to the type of drug [70] or combination of drugs [38], have been reported. The use of a single drug appears to reduce the risk of drug-drug interactions and local side effects, and the risk of infection is avoided if correct ascetic procedures are employed [5]. Literature reports that the subcutaneous infections [6, 71] seem to be caused by external contamination and malpractice rather than to the technique itself.

## 5. Role of Skin Cytochromes

The potential role of skin cytochromes is still under discussion both for the pathway of drugs and local interaction [72–77]. The cytochromes expressed in human keratocytes could influence a number of active compounds available for transdermal administration (analgesics, anti-inflammatories, antibiotics, antifungal, and a large number of products recommended by manufacturers for their anti-aging effects), but there are very little data available on possible drug-drug interactions, metabolic pathway activation, photosensitivity reactions, or other bioactivities [78, 79]. For this reason it is preferable to avoid pharmacological mixtures of drugs, drug combined with herbal medicines or other active substances injected by intradermal route.

## 6. Rationale for Analgesic Drugs Injected at Local Level

A large percentage of patients treated with mesotherapy for musculoskeletal pain disorders had rapid pain relief, generally when the patient responds within the first three sessions of therapy [31–57, 60]. Importantly, in all the studies reviewed in which a wide range of agents (NSAIDs, myorelaxants, EDTA, calcitonin, or vasorelaxants alone or in combination with an anesthetic) were injected at a local level using mesotherapy, no significant adverse events were observed. In these studies a low dose of NSAIDs was frequently used. 

 The World Health Organization (WHO) since 1986 recommends a therapeutic approach for the management of pain based on a three-step process according to the severity of the pain with NSAIDs used in the first step [80]. These drugs exhibit analgesic effects by COX inhibition and the reduction of prostaglandin and other inflammatory mediators [81]. Nitric oxide seems to play a major part in local inflammation [82], and the injection of analgesic drugs (including NSAIDs) may activate neuronal nitric oxide synthase to produce nitric oxide and consequently peripheral antinociception [83]. NSAIDs are useful in a broad range of painful conditions, but renal and gastrointestinal toxicity and cardiovascular complications that occur during the long-term systemic administration constitute a major limitation for their use in patients with chronic pain [84, 85]. Administration of NSAIDs by intradermal injection could represent an alternative therapeutic strategy as lower doses and reduced frequency of administration compared with systemic administration required to obtain the same level of pain control [55]. The drug-sparing benefits of NSAIDs administered by mesotherapy, alone or in combination with other systemic therapies, remain to be quantified. Some authors report that NSAIDs are safe in combination with other agents [55], and several trials investigated NSAIDs and anesthetics given in combination, but it is important to stress that if changes in pH, colour, precipitation, or flocculation are observed it is preferable to avoid the use of the “cocktails” of drugs. In addition, if skin or systemic reactions occur it is not possible to establish which drug is responsible.

In myofascial syndrome where pain is caused by muscle spasm and hyperactivity of muscle fibers, myorelaxants seem to be a rational choice [31–33, 35, 38, 47]. Mechanical, chemical, or heat stimuli in the skin, muscles, and joints are translated into action potential by primary sensory neurons which constitute A*δ* and c-fibers. These primary neurons upregulate opioid receptor expression, and nerve growth factors are released into the peripheral tissues during the inflammatory process. It might be that opioids have a role in pain control during the inflammatory process, and the fact that local administration of opioid-receptors antagonists may exacerbate pain support this thesis [86]. Although morphine is normally given subcutaneously, no clinical data are available when morphine is given intradermally. The intradermal route of administration of morphine is an exciting area to study as it may allow a lower dose to be administered while providing effective pain control.

It still remains to be clarified if the site of injection plays a role. The effects of trigger-point mesotherapy and acupuncture-point mesotherapy (both with lidocaine) were recently compared [56], but large clinical trials are required to better understand the musculoskeletal mechanisms of pain before one method over another can be recommended. Pain relief achieved by myofascial trigger-point injections of bupivacaine was reversed with intravenous naloxone [87], providing clear evidence that the endogenous opioid system is involved in pain reduction at the level of some trigger points and that opioids play a role in the activation of peripheral mechanisms of nociception. It has also been suggested that keratinocytes as well as T lymphocytes are involved in peripheral nociception, but the therapeutic implications of these activities remain to be established [88, 89].

## 7. Recommendations

In 2010, a panel of experts from a range of specialities reviewed and validated the scientific rationale, advantages, indications and contraindications of the use of mesotherapy, with the aim of formulating a series of recommendations on the appropriate use of local intradermal therapy [5]. There was overwhelming consensus among the group members that local intradermal administration is a valuable therapeutic option in the treatment of painful and locoregional conditions, and if used correctly it represents an important addition to the physicians' armamentarium [5]. The experts agreed that mesotherapy should be performed only after a medical diagnosis, and patients should receive information on risk/benefits ratio in particular when off-label drugs are used. They also recommended strongly that the patient's written informed consent should always be obtained before the procedure is carried out to ensure that all possible therapeutic options have been explored and considered. A complete patient clinical report form to include a detailed report of the characteristics of the patient's condition and pharmacological (drugs, doses, and route of administration) and nonpharmacologic techniques previously/currently used together with outcome reports and details of any adverse events observed/reported is mandatory. In this way systematic data can be collected and analyzed retrospectively to determine the efficacy of a given therapeutic approach, and clinical data can be shared among the multidisciplinary team. This procedure has been adopted in Italy since March 2010 where physicians and other healthcare personnel involved have an obligation to record all information in the patient's clinical record. The use of previously untested compounds should be avoided (the exception being clinical trials that conform to good clinical practice, with ethical committee agreement). The physician should supply patients with information in a clear way so that he/she can make an informed decision. All adverse events should be reported to the pharmacovigilance health authorities.

**Figure 1:**
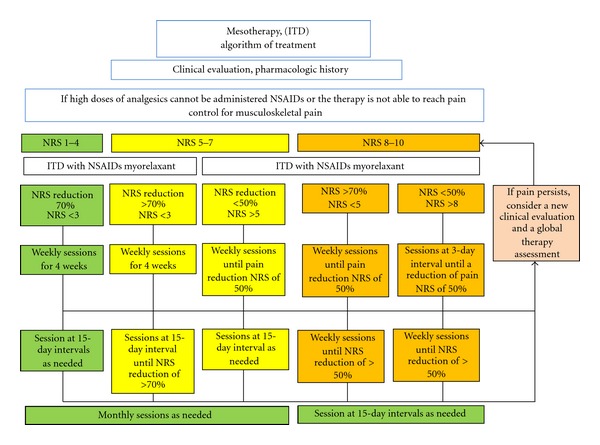
Example of algorithm for the use of NSAIDs and myorelaxant with mesotherapy (intradermal therapy—ITD) in musculoskeletal pain.

The use of a mixture of drugs did not receive full consensus among experts due to the increased risk of pharmacological interactions, even if in some circumstances two active ingredients have been reported to be safe (there are no data on possible drug interactions, although changes in pH, color, and precipitation/flocculation have been observed in mixtures of drugs. Moreover, using drug mixtures, it is not possible to identify the effects of individual drugs both in terms of efficacy and tolerability). Particular attention should be paid to conforming to international hygiene standards and to avoid contamination of the sterile syringes and needles. Before using intradermal therapy, or other therapies, an internationally validated scale should be used to classify pain based on the type and intensity. Mesotherapy is recommended in the management of musculoskeletal pain alone or in combination with other therapies where there are no contraindications to the pharmacologically active agents. Mesotherapy is not recommended in patients with a history of allergic reactions, disorders of coagulation (haemophiliacs, undergoing therapy with anticoagulants or antiplatelet agents), pregnant or lactating women, or cancer patients undergoing chemotherapy. Due to the broad range of pain mechanisms and the lack of a recognized standard of care, when mesotherapy is used the patient's response drives subsequent clinical decisions, and the number of sessions depends on the severity of the pain. Mesotherapy protocols allow for one or more cycles of treatment depending on the symptoms and severity of the underlying condition and the individual patient's response to therapy (Figure 1). When treating a chronic painful condition there are normally three distinct phases. The first period (attack) to reduce pain, a second (control) period to confirm the results and to improve efficacy/tolerability ratio, and a subsequent (maintenance) period to prevent recurrences [4]. In real-life clinical practice, we propose an example of algorithm to manage patients who can benefit from a local treatment instead of a systemic more aggressive pharmacologic approach or who can benefit from a synergistic combination of local and systemic therapy. Obviously, every physician who applies mesotherapy for analgesic purposes should assess pain before and during the treatment. For this reason we strongly suggest the use of validated scales to assess pain and to consider extending the mesotherapy treatment only if the patient declares a relief of at least 50% from baseline.

## 8. Conclusions

For many years, mesotherapy has been considered by physicians (and nonmedical personnel) as a “personal” pharmacological approach, treatment practices were based on personal observations and experience, but in the era of evidence-based medicine this approach is no longer adequate, and standard treatment algorithms are required. We have reviewed and analyzed the data available in the treatment of various forms of musculoskeletal pain, and although the number of randomized, controlled trials is still low, overall results suggest that this technique provides clinical benefits and importantly is well tolerated. Mesotherapy allows the drug to diffuse slowly into the tissues at the site of injection and to have a prolonged period of action at low dosages. Microinjections facilitate the rebalancing of the nociceptive system through a series of complex and as yet not well-understood local actions involving nociceptive receptors, nociceptive central feedback mechanisms, and the immune system. The pain relief reported by patients occurs as a result of all these phenomena.

The Italian Society of Mesotherapy considers that the intradermal injection of drugs is effective and well tolerated if administered according to approved techniques and when administered after a complete clinical workup from which there is a clear rationale for local treatment. We believe that this method provides potential benefits in terms of clinical effectiveness and cost savings in the management of musculoskeletal pain. Many studies have been conducted in the open, and few randomized controlled trials have a limited number of subjects, it should be pointed out however that more large-scale and randomized clinical trials are required to confirm its efficacy and tolerability in general, and in particular we have identified two important areas for further reach: first to confirm the efficacy of intradermal NSAIDs in localized pain to reduce the risk of the known systemic effects of this class of drugs and second the use of intradermal opioids in order to improve our understanding of how to extend the effectiveness of these analgesics in musculoskeletal painful conditions.

## Figures and Tables

**Table 1:** Tissue levels of Na-ketoprofen (*μ*g) detected by chromatography following local intradermal (LIT) or intramuscular (IM) administration, modified with permission from [23].

Time (hours)	Skin		Muscle		Articular tissue	
	LIT	IM	LIT	IM	LIT	IM
0	nd	nd	nd	nd	nd	nd
0.5	124.9	4.8	1.5	33.3	25.8	18.6
1	42.3	t	3	t	19.3	0.3
2	15.6	t	23.8	t	10.8	t
4	10.9	t	19.3	t	8.3	t
7	t	nd	100.6	t	6.2	t
10	t	nd	102.2	t	7	t
24	nd	nd	14.9	nd	0.8	nd

nd = not detectable; t = trace.

**Table 2:** Initial open studies in patients with musculoskeletal pain.

Study	Disease	No. of patients	Study characteristics	Drugs utilized	Control	Period of followup	No. of sessions	Outcomes
Ruggeri et al. 1981 [31]	Cervico-dorsal rachialgia, lumbosacral spinalgia, coxarthrosis, gonarthrosis, and Duplay's disease	984	M R	NSAIDs, myorelaxants, and procaine	NC	from 3 to 6 weeks	1–3 sessions at 1- or 2- week intervals	80% of pts reported pain reduction. More effective in cervicodorsal rachialgia (87%), tendinitis and bursitis (83.3%); coxarthrosis (50%)

Colombo et al. 1981 [32]	Acute cervicalgia, lumbar pain, acute myositis, tendinitis, traumatic disorders, shoulder-hand syndrome	484	M P O	Vasodilators, NSAIDs, myorelaxants, and procaine	NC	9 days	3 sessions of mesotherapy at 3-day intervals	Pain reduction in 83.6% of pts.

Saraceni et al. 1981 [33]	Periarthritis, rachialgia, gonarthrosis, tendinitis, epitrocleitis, and carpal tunnel syndrome	200	M P O	NSAIDs, myorelaxant, vasodilator, and anaesthetic	NC	3 sessions of treatment in 21 days	3 sessions of treatment in 21 days	Pain reduction in 91% of pts.

Piantoni et al. 1981 [34]	Osteoarticular disorders with pain (cervical, dorsal, lumbar column, shoulder, hip, and knee)	46	P O	NSAIDs	NC	21 days	sessions of treatment in 21 days	Pain reduction in 78% of pts.

Pezone et al. 1981 [35]	Osteoarticular disorders	256	M R	NSAIDs, myorelaxant, vasodilator, and anaesthetic	NC	30 days	sessions every 3 days	Pain reduction in 52.7 % and improvement of articular function in 54.7% of pts.

Currò and Bearzato 1981 [36]	Gonarthrosis	20	P O	s-adenosil l-methionine + lidocaine	NC	7 weeks	6 sessions	Pain reduction in 90% of pts.

Guazzetti et al. 1988 [37]	Musculoskeletal affections	15	P O	Naproxene, procaine	NC	?	from 3 to 9 mesotherapy	Positive results in 90,5% of pts

Narvarte and Rosset-Llobet 2011 [38]	Osteomuscular disorders	67	P O	Thiocolchicoside diazepam buflomedil piroxicam	NC	4 weeks	from 1 to 18 sessions	Positive efficacy/safety

Capone et al. 1994 [39]	Calcific tendinitis of the shoulder	50	P O C	Disodium EDTA	Mesotherapy versus ionophoresis	24 months	nr	Positive effects with both techniques (80% of patients)

Piantoni et al. 1985 [40]	Osteoporosis	1	CR	Calcitonine	NC	30 days	2 sessions per week	Pain reduction

Currò and Bearzatto 1985 [41]	Postherpetic neuritis	7	P O	NSAIDs and procaine	NC	7 weeks	weekly sessions	Pain reduction in 57% of pts after the first session

Currò et al. 1983 [42]	Degenerative arthrosis in a new acute stage of pain	96	P O	NSAIDs, s-adenosil l-methionine, and procaine	NC	1 year	6 sessions for 1, 2, or 3 cycle in a year	Reduction of pain, drugs consumption (67%), and absences from work (30%)

Biondi et al. 1985 [43]	Tendinitis, scapulohumeral periarthritis	44	P C	Superoxide dismutase (SOD) and mepivacaine	Mepivacaine	5 months	3–6 sessions every 4–8 days	Pain reduction in 90% of pts or recovery in the SOD+anesthetic group versus 33% in the group with the anesthetic alone

Pezone et al. 1986 [44]	Osteoporosis/arthritis	32	P O	Calcitonine	NC	2–10 weeks	weekley sessions	Pain reduction (76.5% of pts) in particular in pts with osteoporosis and arthritis

Solinas et al. 1987 [45]	Tendinopathies	20	P O	Ergoteine	NC	nr	nr	Combination with laser therapy more effective (in term of pain reduction) than traditional therapies

Garzya et al. 1987 [46]	Muscular skeletal pain	100	P O C	NSAIDs and anesthetic	Naproxen, lysine acetylsalicylate, and ketoprofen	52 days	3 session at weekley intervals	Pain reduction; no differences between NSAIDs evaluated. Pts with cervicodynia and gonalgia received better clinical benefits

The table lists clinical studies or case reports to evaluate the reduction of pain in various clinical conditions. The pain was noted with visual scales.

M: multicentric, R: retrospective, P: prospective, O: open, C: controlled, NC: noncontrolled, CS: case report, and nr: nonreported.

**Table 3:** Clinical studies conducted in athletes both professional and amatory with posttraumatic pain.

Study	Disease	No. of patients	Study characteristics	Drugs utilized	Control	Period of followup	No. of sessions	Outcome
Cereser et al. 1985 [47]	Pain posttraumatic in rugby professional players	133	R O	NSAIDs, myorelaxant, vasorelaxant, and mepivacaine	NC	up to 4 months	1–4 sessions	Pain reduction and functional recovering of sporting competitive activity in shorter time then conventional therapies

Gribaudo et al. 1982 [48]	Pubic myoenthesitis	256	P O	NSAIDs and vasorelaxant	NC	6 months	from 2 to 5 sessions at 10–20-day intervals	Complete functional recovery after 4 sessions

Lepore and Savino 1983 [49]	Acute lumbosciatic pain in athletes	20	P O	Neuramidium, Procaine	NC	4 months	2–6 sessions	Pain reduction and functional recovery in 90% of pts

Gribaudo et al. 1986 [50]	Patellar tendonitis	126	P O	Superoxide dismutase (SOD), lidocaine, and vasorelaxant	NC	1 month	weekley sessions	85% of pts reach complete pain relief (form 1 to 4 sessions)

Gribaudo et al. 1986 [51]	Ileo-tibial band friction syndrome	40	P O	NSAIDS, vasorelaxant, and anesthetic	NC	3 months	weekly sessions	Pain relief in 55% of pts after 2 sessions; 97.5% after 3 sessions

Gribaudo et al. 1987 [52]	Myonthesitis of the leg	203	P O	NSAIDs, vasorelaxant, and lidocaine	NC	2 months	sessions at 7-8-day intervals	60.8% of pts reach complete recovery with 1 session; 96.6% of pts reach complete recovery with 3 sessions. Mesotherapy was more efficacy in pts with recent pain.

The table lists clinical studies to evaluate the reduction of pain in various clinical conditions. The pain was noted with visual scales.

R: retrospective, P: Prospective, O: open, and NC: noncontrolled.

**Table 4:** Randomized, controlled clinical trial in patients with low back pain, cervicobrachialgia and calcific painful tendinitis of the shoulder.

Study	Disease	No. of patients	Study characteristics	Drugs utilized	Control	Period of followup	No. of sessions	Outcome
Parrini et al. 2002 [53]	Acute lumbosciatic pain syndrome	44	RA	Acetylsalicylic acid	PC	1 day	1	Pain reduction/safety

Monticone et al. 2004 [54]	Low back pain (sacroiliac dysfunction)	22	RA P C	NSAIDs	Laser therapy	1 year	2 session per week (8 sessions)	Pain reduction better for mesotherapy, exercise and dynamic support than laser therapy

Costantino et al. 2010 [55]	Low back pain	84	RA P C	Lidocaine, ketoprofen, and methylprednisolone	Standard therapy ketoprofen, esomeprazole and methylprednisolone	6 months	5 sessions	Same efficacy and safety systemic therapy

Di Cesare et al. 2010 [56]	Low back pain	62	RA P C	Lidocaine	Mesotherapy in acupuncture points versus mesotherapy in trigger points	12 weeks	4 sessions	Better reduction of pain with mesotherapy in acopunture points

Cacchio et al. 2009 [57]	Calcific tendinitis of the shoulder	80	RA DB	Disodium EDTA and procaine	PC	1 year	1 session at weekly intervals for 3 weeks	Calcification disappeared completely in 62.5% and partially in 22.5% of pts; partially effects were registered in 15% of pts in the control group

Palermo et al. 1991 [60]	Cervicobrachialgia	20	RA P O C	Lidocaine and myorelaxant	TENS	20 days	6 TENS 4 mesotherapy	Mesotherapy combined with TENS improves symptoms management, and reduces the number of needed TENS sessions

The table lists clinical studies or case reports to evaluate the reduction of pain in various clinical conditions. The pain was noted with visual scales.

P: prospective, O: open, DB: double blind, RA: randomized, C: controlled, and PC: placebo controlled.

## References

[1] Pistor M (1976). What is mesotherapy?. *Le Chirurgien-Dentiste de France*.

[2] Dalloz-Bourguignon A (1979). A new therapy against pain: mesotherapy. *Journal Belge de Medecine Physique et de Rehabilitation*.

[3] Rohrich RJ (2005). Mesotherapy: what is it? Does it work?. *Plastic and Reconstructive Surgery*.

[4] Maggiori S (2004 ). *Manuale di Intradermoterapia Distrettuale. La Mesoterapia in Italia*.

[5] Mammucari M, Gatti A, Maggiori S, Bartoletti CA, Sabato AF (2011). Mesotherapy, definition, rationale and clinical role: a consensus report from the italian society of mesotherapy. *European Review for Medical and Pharmacological Sciences*.

[6] Herreros FOC, de Moraes AM, Velho PENF (2011). Mesotherapy: a bibliographical review. *Anais Brasileiros de Dermatologia*.

[7] Sarkar R, Garg VK, Mysore V (2011). Position paper on mesotherapy. *Indian Journal of Dermatology, Venereology and Leprology*.

[8] Atiyeh BS, Ibrahim AE, Dibo SA (2008). Cosmetic mesotherapy: between scientific evidence, science fiction, and lucrative business. *Aesthetic Plastic Surgery*.

[9] Merkey H, Bogduk N (1994). *Classification of Chronic Pain: Descriptions of Chronic Pain Syndromes and Definition of Pain Terms*.

[10] Mannion RJ, Woolf CJ (2000). Pain mechanisms and management: a central perspective. *Clinical Journal of Pain*.

[11] Sabato AF (2010). Idiopathic break through pain: a new hypothesis. *Clinical Drug Investigation*.

[12] Wang W, Wang W, Mei X (2009). Crosstalk between spinal astrocytes and nuerons in nerve injury-injuced neuropathic pain. *Plos ONE*.

[13] Turk DC, Okifuji A, Fishman SM, Ballantyne JC, Rathmell JP (2009). Pain terms and taxonomies of pain. *Bonica’s Management of Pain*.

[14] Freynhagen R, Baron R, Tölle T (2006). Screening of neuropathic pain components in patients with chronic back pain associated with nerve root compression: a prospective observational pilot study (MIPORT). *Current Medical Research and Opinion*.

[15] Turk DC, Wilson HD, Cahana A (2011). Treatment of chronic non-cancer pain. *The Lancet*.

[16] Woolf AD, Zeidler H, Haglund U (2004). Musculoskeletal pain in europe: its impact and a comparison of population and medical perceptions of treatment in eight european countries. *Annals of the Rheumatic Diseases*.

[17] Katz JD, Shah T (2009). Persistent pain in the older adult: what should we do now in light of the 2009 american geriatrics society clinical practice guideline?. *Polskie Archiwum Medycyny Wewnetrznej*.

[18] Crosby J (2009). Osteoarthritis: managing without surgery. *Journal of Family Practice*.

[19] Mason L, Moore RA, Edwards JE, Derry S, McQuay HJ (2004). Topical nsaids for chronic musculoskeletal pain: systematic review and meta-analysis. *BMC Musculoskeletal Disorders*.

[20] Atiyeh BS, Ibrahim AE, Dibo SA (2008). Cosmetic mesotherapy: between scientific evidence, science fiction, and lucrative business. *Aesthetic Plastic Surgery*.

[21] Rotunda AM (2009). Injectable treatments for adipose tissue: terminology, mechanism, and tissue interaction. *Lasers in Surgery and Medicine*.

[22] Koivisto VA, Felig P (1980). Alterations in insulin absorption and in blood glucose control associated with varying insulin injection sites in diabetic patients. *Annals of Internal Medicine*.

[23] Binaglia L, Marconi P, Pitzurra M (1981). Absorption of Na ketoprofen administered intradermally. *Giornale di Mesoterapia*.

[24] Binaglia L, Marconi P, Pitzurra M (1981). The diffusion of intradermally administered procaine. *Giornale di Mesoterapia*.

[25] Pitzurra M, Cavallo R, Farinelli S, Sposini T, Cipressa T, Scaringi L (1981). On the intradermal inoculation of antibiotics: some experimental data. *Giornale di Mesoterapia*.

[26] Pitzurra M, Marconi P (1981). Immunogenesis and mesotherapy: the immunoresponse to antigens inoculated intradermally. *Giornale di Mesoterapia*.

[27] Coudeville L, Andre P, Bailleux F, Weber F, Plotkin S (2010). A new approach to estimate vaccine efficacy based on immunogenicity data applied to influenza vaccines administered by the intradermal or intramuscular routes. *Human Vaccines*.

[28] Hsu CC, Kuo HC, Hsu CT, Gu Q (2011). Abdominal mesotherapy injection extended the absorption of follicle-stimulating hormone. *Fertility and Sterility*.

[29] Crenna P, Mancia P (1981). Reflex actions in mesotherapy. *Giornale di Mesoterapia*.

[30] Sticchi L, Alberti M, Alicino C, Crovari P (2010). The intradermal vaccination: past experiences and current perspectives. *Journal of Preventive Medicine and Hygiene*.

[31] Ruggeri F, Bartoletti CA, Maggiori S (1981). Clinical results of the multicentric experimentation. *Giornale di Mesoterapia*.

[32] Colombo I, Cigolini M, Combi F (1981). Clinical results of the multicentric experimentation. *Giornale di Mesoterapia*.

[33] Saraceni V, Palieri G, De Pedis M (1981). Clinical results of the multicentric experimentation. *Giornale di Mesoterapia*.

[34] Piantoni D, Cotichelli E, Di Gianvito P (1981). Clinical results of the multicentric experimentation. *Giornale di Mesoterapia*.

[35] Pezone A, Villa L, Martini D (1981). Clinical results of the multicentric experimentation. *Giornale di Mesoterapia*.

[36] Currò F, Bearzato A (1981). Use of the S-adenosil l-methionine (Same) in the treatment of degenerative arthropathies of arthrosis. *Nature*.

[37] Guazzetti R, Iotti E, Marinoni E (1988). Mesotherapy with naproxin sodium in musculoskeletal diseases. *Rivista Europea Per Le Scienze Mediche E Farmacologiche*.

[38] Narvarte DA, Rosset-Llobet J (2011). Safety of subcutaneous microinjections (mesotherapy) in musicians. *Medical Problems of Performing Artists*.

[39] Capone M, Stancati MT, Tolla V, Chiatti R, Muscolo V, Pasquale M (1994). Observations on the administration of sodium edetate in calcified scapulohumeral periarthritis. Ionophoresis and mesotherapy: comparison of two techniques. *Ortopedia e Traumatologia Oggi*.

[40] Piantoni D, Cotichellil E, Santilli W (1985). Use of calcitonin in regional osteoporosis. *Giornale di Mesoterapia*.

[41] Currò F, Bearzatto A (1985). Mesotherapy in the treatment of post-zoster neuritis. *Giornale di Mesoterapia*.

[42] Currò F, Bearzatto A, Fontanini C (1983). Mesotherapy in a general medicine department: a year of activity. *Giornale di Mesoterapia*.

[43] Biondi G, Romano M, Marcone E, Concetta Meo M, Attanasio L (1985). Orgotein: our experience in rheumatic pathology. *Giornale di Mesoterapia*.

[44] Pezone A, Santuari E, Villa ML (1986). The distinct analgesic action of calcitonin in treating painful diseases of joints with mesotherapy. *Giornale di Mesoterapia*.

[45] Solinas G, Solinas AL, Perra P, Solinas FL (1987). Treatment of mechanical tendinopathies by mesotherapy with orgotein in combination with laser therapy. *Riabilitazione*.

[46] Garzya G, Leucci PF, Greco T, Branca L, Moschettini V (1987). Comparative stydy of three non-steroid antiinflammatory drugs used with mesotherapy techique in 100 geriatric patients affected by muscular -skeletal pathology. *Giornale di Mesoterapia*.

[47] Cereser C, Ganzit GP, Gribaudo C (1985). Injuries affecting the locomotory system during the game of rugby. Reports of 133 cases treated with mesotherapy. *Giornale di Mesoterapia*.

[48] Gribaudo CG, Ganzit GP, Astegiano P (1982). Mesotherapy in treating pubic myoenthesitis. *Giornale di Mesoterapia*.

[49] Lepore F, Savino V (1983). Acute lumbo sciatic pain in athletes. *Giornale di Mesoterapia*.

[50] Gribaudo CG, Ganzit GP, Astegiano P, Canata GL (1986). Mesotherapy in treatment of the ileo-tibial band friction syndrome. *Giornale di Mesoterapia*.

[51] Gribaudo CG, Ganzit GP, Canata GL, Gerbi G (1986). Patellar tendonitis: treatment with ergotein in mesotherapy. *Giornale di Mesoterapia*.

[52] Gribaudo CG, Canata GL, Ganzit GP, Gerbi G (1987). Mesotherapy in the treatment of myoenthesitis ofthe leg in athletes. *Giornale di Mesoterapia*.

[53] Parrini M, Bergamaschi R, Azzoni R (2002). Controlled study of acetylsalicylic acid efficacy by mesotherapy in lumbo-sciatic pain. *Minerva Ortopedica E Traumatologica*.

[54] Monticone M, Barbarino A, Testi C, Arzano S, Moschi A, Negrini S (2004). Symptomatic efficacy of stabilizing treatment versus laser therapy for sub-acute low back pain with positive tests for sacroiliac dysfunction: a randomised clinical controlled trial with 1 year follow-up. *Europa Medicophysica*.

[55] Costantino C, Marangio E, Coruzzi G (2011). Mesotherapy versus systemic therapy in the treatment of acute low back pain: a randomized trial. *Evidence-Based Complementary and Alternative Medicine*.

[56] Di Cesare A, Giombini A, Di Cesare M, Ripani M, Vulpiani MC, Saraceni VM (2011). Comparison between the effects of trigger point mesotherapy versus acupuncture points mesotherapy in the treatment of chronic low back pain: a short term randomized controlled trial. *Complementary Therapies in Medicine*.

[57] Cacchio A, De Blasis E, Desiati P, Spacca G, Santilli V, De Paulis F (2009). Effectiveness of treatment of calcific tendinitis of the shoulder by disodium EDTA. *Arthritis Care and Research*.

[58] Gazzi A, Ponzetti F, Ricci L (1984). Mesotherapy with edetic acid in calcified humeroscapular periarthritis (Duplay's disease). Encouraging results. *Riabilitazione*.

[59] Soncini G, Costantino C (1998). The treatment of pathologic calcification of the shoulder tendons with EDTA bisodium salt by mesotherapy. *Acta Biomed Ateneo Parmense*.

[60] Palermo S, Riello R, Cammardella MP (1991). TENS+ mesotherapy association in the therapy of cervico-brachialgia: preliminary data. *Minerva Anestesiologica*.

[61] Colombo I, Cigolini M (1981). An interesting therapeutically synergism: mesotherapy and laser. *Giornale di Mesoterapia*.

[62] Santilli V, Di Girolamo G, Finucci S, Capici S, Paris E (1999). Back pain: low back pain model, treatment with physical and injective therapy. *Rivista di Neurobiologia*.

[63] Médioni G (1980). Results of 6 years of treatment of painful periodontal episodes by mesotherapy. *Le Chirurgien-Dentiste de France*.

[64] Vaillant P (1986). Remission of painful oro-dental symptoms using treatment with mesotherapy. *Le Chirurgien-Dentiste de France*.

[65] Einholtz B, Maudet D, Bicheron M (1990). Use of nhai via mesotherapy in oral surgery. *Actualites Odonto-Stomatologiques*.

[66] Ceccarelli M, Bassano P, Serra ViscontiA, Bartoletti CA (1987). Concerning pain caused by the action of mesotherapy: proposal of a pharmacological buffer. *Giornale di Mesoterapia*.

[67] Serafini G, Marineo G, Sabato AF (2000). "scrambler therapy": a new option in neuropathic pain treatment?. *Pain Clinic*.

[68] Marineo G, Spaziani S, Sabato AF, Marotta F (2003). Artificial neurons in oncological pain: the potential of Scrambler Therapy to modify a biological information. *International Congress Series*.

[69] Sabato AF, Marineo G, Gatti A (2005). Scrambler therapy. *Minerva Anestesiologica*.

[70] Maggiori E, Bartoletti CA, Maggiori S, Tomaselli F, Dorato D (2010). Local intradermotherapy (ITD) with mesoglicano in PEFS and IVLC, retrospective study. *Trends in Medicine*.

[71] Carbonne A, Brossier F, Arnaud I (2009). Outbreak of nontuberculous mycobacterial subcutaneous infections related to multiple mesotherapy injections. *Journal of Clinical Microbiology*.

[72] Du L, Hoffman SMG, Keeney DS (2004). Epidermal CYP2 family cytochromes P450. *Toxicology and Applied Pharmacology*.

[73] Swanson HI (2004). Cytochrome P450 expression in human keratinocytes: an aryl hydrocarbon receptor perspective. *Chemico-Biological Interactions*.

[74] Du L, Neis MM, Ladd PA, Lanza DL, Yost GS, Keeney DS (2006). Effects of the differentiated keratinocyte phenotype on expression levels of CYP1-4 family genes in human skin cells. *Toxicology and Applied Pharmacology*.

[75] Du L, Neis MM, Ladd PA, Keeney DS (2006). Differentiation-specific factors modulate epidermal cyp1-4 gene expression in human skin in response to retinoic acid and classic aryl hydrocarbon receptor ligands. *Journal of Pharmacology and Experimental Therapeutics*.

[76] Pavek P, Dvorak Z (2008). Xenobiotic-induced transcriptional regulation of xenobiotic metabolizing enzymes of the cytochrome P450 superfamily in human extrahepatic tissues. *Current Drug Metabolism*.

[77] Neis MM, Wendel A, Wiederholt T (2010). Expression and induction of cytochrome P450 isoenzymes in human skin equivalents. *Skin Pharmacology and Physiology*.

[78] Swanson HI (2004). Cytochrome P450 expression in human keratinocytes: an aryl hydrocarbon receptor perspective. *Chemico-Biological Interactions*.

[79] Sanderson JP, Naisbitt DJ, Park BK (2006). Role of bioactivation in drug-induced hypersensitivity reactions. *Aaps Journal*.

[80] World Health Organization (1986). *Cancer Pain Relief*.

[81] Gøtzsche P (2002). Non-steroidal anti-inflammatory drugs. *Clinical Evidence*.

[82] Wahl SM, McCartney-Francis N, Chan J, Dionne R, Ta L, Orenstein JM (2003). Nitric oxide in experimental joint inflammation: benefit or detriment?. *Cells Tissues Organs*.

[83] Romero TRL, Resende LC, Duarte IDG (2011). The neuronal NO synthase participation in the peripheral antinociception mechanism induced by several analgesic drugs. *Nitric Oxide*.

[84] Ambrosio F, Finco G, Mattia C (2006). Siaarti recommendations for chronic non-cancer pain. *Minerva Anestesiologica*.

[85] McGettigan P, Henry D (2011). Cardiovascular risk with non-steroidal anti-inflammatory drugs: Systematic review of population-based controlled observational studies. *PLoS Medicine*.

[86] Stein C, Lang LJ (2009). Peripheral mechanisms of opioid analgesia. *Current Opinion in Pharmacology*.

[87] Fine PG, Milano R, Hare BD (1988). The effects of myofascial trigger point injections are naloxone reversible. *Pain*.

[88] Khodorova A, Navarro B, Jouaville LS (2003). Endothelin-B receptor activation triggers an endogenous analgesic cascade at sites of peripheral injury. *Nature Medicine*.

[89] Verma-Gandhu M, Bercik P, Motomura Y (2006). CD4^+^ T-cell modulation of visceral nociception in mice. *Gastroenterology*.

